# 

**Published:** 1887-10

**Authors:** 


### CONTENTS, OCT. 1887, (VOL. III, NO. IV.)

ORIGINAL COMMUNICATIONS—Notes of the Washington Congress: by R. B. White, M. D., Ennis, Texas.—Was it Pulmonary Embolism? By A. J. Gray, M. D., Martin's Mills.—A case of Strangulated Inguinal Hernia, in an inmate of the State Lunatic Asylum: By A. N. Denton, M. D., late Superintendent, etc., Austin.............. 122–132

SOCIETY NOTES—Proceedings of the Terrell Medical Society.—American Public Health Ass'n, 16th Annual Session.—Dr. N. S. Davis to Dr. Q. C. Smith.—East Texas Medical Ass'n.—Austin District Medical Society Physicians' Mutual Benefit Ass'n of Texas.—Thanks: Still Organizing: Dr. White's Paper................. 134–138

CORRESPONDENCE—Letter from Dr. F. T. Paine on Electrical treatment of Diseases....................... 139

CULLINGS FROM CONTEMPORARIES—The New Rival of Cocaine.—A Fable.—A New Morbific Germ Discovered. 143–144

EDITORIAL—The Cholera in New York.—Death of Dr. James A. Gray.—Dr. Walker's Paper on Hernia..... 146-152

MEDICAL NEWS AND MISCELLANY....................... 153

NECROLOGICAL—Dr. Andrew Robert Kilpatrick. (Portrait frontispiece)...................................... 159

BIBLIOGRAPHY—Physicians Visiting List for '88.—Index Catalogue S. G. O.—Practical Urine Testing.—Annals of Gynecology.................................. 163

ADVERTISERS' NOTICES—Numerous..................... 164

